# Supplementary figures and images for: Mesenchymal stromal cell activation by breast cancer secretomes in bioengineered 3D microenvironments
Source: Life Sci Alliance. 2019 Jun 3;2(3):e201900304. doi: 10.26508/lsa.201900304 (PMC6549139; doi:10.26508/lsa.201900304)

Source data for Figure 1F

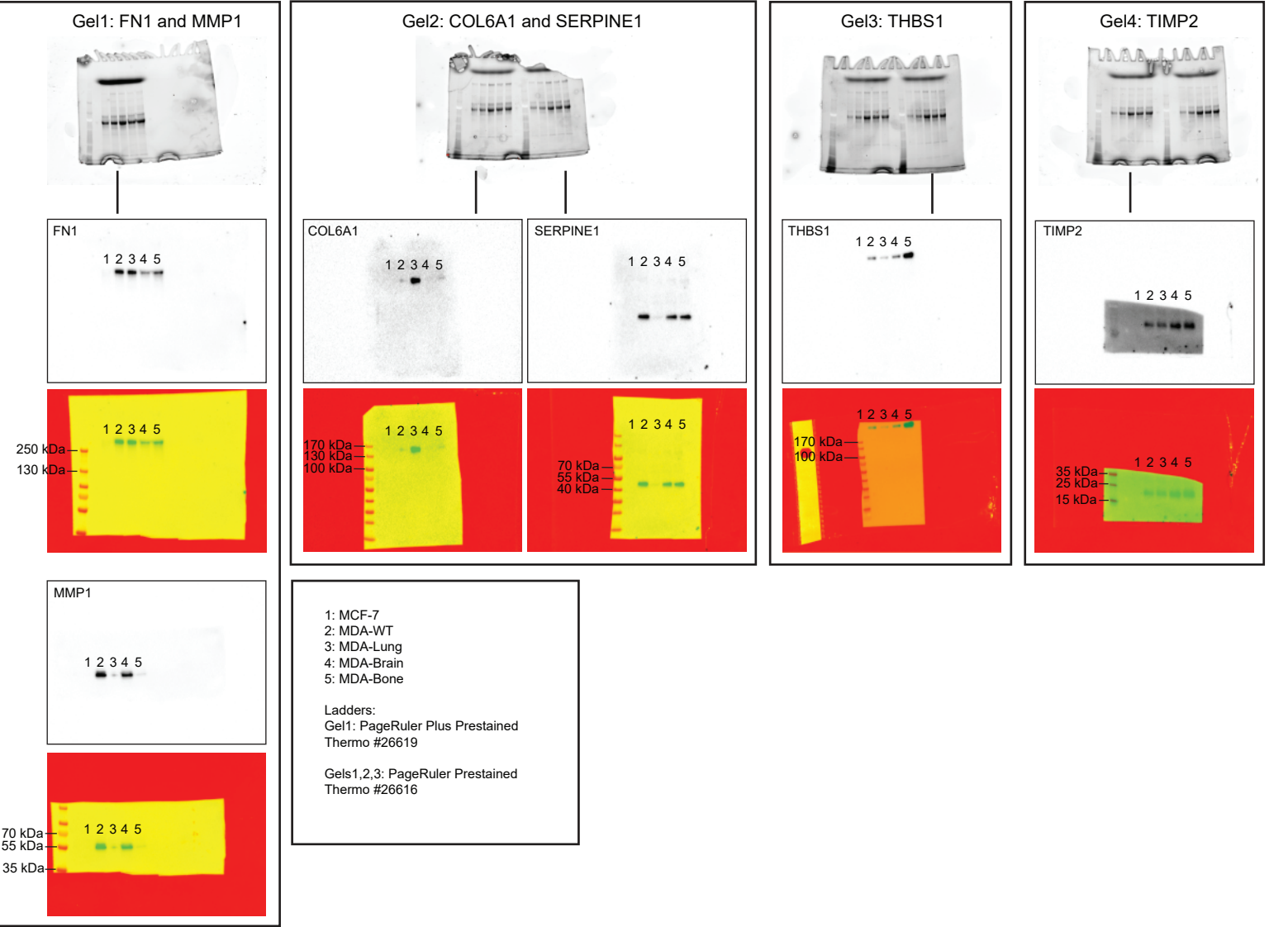

Supplement: Supplementary file 2 [file LSA-2019-00304_SdataF1.pdf]
